# Impact of high-protein, low-calorie diet on anthropometric indices, glycation, and inflammation associated with the fat mass and obesity (FTO) gene among individuals with overweight/obesity

**DOI:** 10.3389/fnut.2025.1619543

**Published:** 2025-10-20

**Authors:** Bibi Hajira, Bismillah Sehar, Fakhrunisa, Sami Siraj, Muhammad Omar Malik, Huma Naqeeb, Ali Madi Almajwal, Sharifa AlBlooshi, Hayder Hasan, Iftikhar Alam, Falak Zeb, Fahad Lodhi

**Affiliations:** ^1^Department of Human Nutrition, Institute of Basic Medical Sciences, Khyber Medical University, Peshawar, Pakistan; ^2^Faculty of Health, Education and Life Sciences, Birmingham City University, Birmingham, United Kingdom; ^3^Institute of Pharmaceutical Sciences, Khyber Medical University, Peshawar, Pakistan; ^4^Department of Physiology, Institute of Basic Medical Sciences, Khyber Medical University, Peshawar, Pakistan; ^5^Human Nutrition and Dietetics Department Women University Mardan, Mardan, Pakistan; ^6^Department of Community Health Sciences, College of Applied Medical Sciences, King Saud University, Riyadh, Saudi Arabia; ^7^College of Natural and Health Sciences, Zayed University, Dubai, United Arab Emirates; ^8^Department of Clinical Nutrition and Dietetics, College of Health Sciences, University of Sharjah, Sharjah, United Arab Emirates; ^9^Nutrition and Food Research Group, Research Institute of Medical and Health Sciences, University of Sharjah, Sharjah, United Arab Emirates; ^10^Department of Human Nutrition and Dietetics, Bacha Khan University Charsadda, Charsadda, Pakistan; ^11^Department of Public Health and Behavioral Sciences, Dubai Medical College for Girls, Dubai Medical University, Dubai, United Arab Emirates

**Keywords:** obesity, CML, high protein low calorie diet, FTO gene, anthropometrics

## Abstract

**Background:**

The common polymorphism rs9939609 of the fat mass and obesity gene (FTO) has been associated with increased susceptibility to obesity, but this association appears to be modified by diet. High protein diets have been shown to reduce weight and may increase the formation of circulating advanced glycation end products (AGEs). Obesity, on the other hand, is also associated with increased formation of AGEs, leading to oxidative stress and inflammation.

**Objectives:**

This study was designed to investigate the impact of a high-protein and low-calorie (HPLC) diet on anthropometric indices and circulating AGEs levels associated with the FTO variant rs9939609 among overweight/obese individuals.

**Methods:**

In this interventional study, 60 overweight and obese individuals (aged 18–50 years) with no comorbidity were assigned to an HPLC diet of 800 kcal and ~100 g protein/day for 4 weeks. The enrolled participants were divided into three groups (each group, *n* = 20) based on FTO genotyping, i.e., AA, TT, and AT, using whole blood samples. Body mass index (BMI), waist circumference (WC), hip circumference (HC), and waist-to-hip ratio (WHR) were measured before and after intervention. Serum analysis of carboxymethyl lysine (CML) and interleukin-6 (IL-6) was performed at baseline (day 0) and at the endline (day 28).

**Results:**

The weight (*p* = 0.01), WC (*p* = 0.002), and WHR (*p* = 0.04) were significantly different among the three genotypes. The risk allele group (AA) had a higher mean weight (95.74 ± 19.13 kg), WC (105.85 ± 14.55 cm), and WHR (0.93 ± 0.08) compared with the wild-type TT. HPLC diet significantly decreased weight (*p* = 0.02), BMI (*p* = 0.03), WC (*p* < 0.001), and WHR (*p* = 0.02), while no significant effect was found on CML and IL-6 in all three genotypes at the end of intervention. The effect size estimates indicated significant variation explained by the FTO gene in weight (η^2^ = 0.158), BMI (η^2^ = 0.114), WC (η^2^ = 0.235), and WHR (η^2^ = 0.138).

**Conclusion:**

This study concludes that an HPLC diet modifies the variation of the FTO rs9939609 genotype and anthropometric measurements. These findings also suggest that high dietary protein intakes may protect against the obesogenic effects of FTO risk genotypes, leading to weight loss and improved metabolic parameters.

## 1 Introduction

Obesity is an inflammatory and pathological condition that affects various physiological functions and poses a threat to the health of individuals (1, 2). It occurs due to positive energy balance, i.e., when energy consumption is greater than energy expenditure (3). A large segment of the world population is affected by obesity, including both adults and children, irrespective of the geographical locality, ethnicity, or socioeconomic status. However, developing countries are more susceptible to obesity (4, 5). Over the past four decades, the global burden of obesity has tripled, affecting more than 1.9 billion (39%) adults (18 years and older) (5). Among developing countries, Pakistan is also facing a rising prevalence of obesity (5.1%) and overweight (22.8%) (6). The risk factors include economic growth, availability of poor-quality and low-cost foods, industrialization, urbanization, and environmental influences such as a westernized dietary pattern and sedentary lifestyle, all of which contribute to the high prevalence of obesity worldwide (7–9). Genetic predisposition also plays an important role in the development of obesity, accounting for 40–60% of the variation in body mass index (BMI) (7, 9). Obesity is related to chronic diseases such as diabetes, hypertension, cardiovascular diseases, and some cancers. In addition, it increases the risk of disability (10, 11).

Genetic polymorphism explores the pathogenesis and diversity of diseases and the nature of phenotype diversity (12). Genome-wide association made it possible to identify several predisposed loci for a complex trait of a disease (9). For instance, the fat mass and obesity gene (FTO) gene variation is found to be associated with genes predisposing to obesity, with increased fat accumulation in the body by influencing energy balance, appetite regulation, and metabolism (13). Genome-wide association studies (GWAS) have identified single-nucleotide polymorphisms (SNPs) within the FTO gene to be associated with human body fat mass (9). FTO SNP rs9939609 has been proven within different populations, including the South-Asian ethnicity, to be strongly related to various indices of obesity (13–16). Frequency of FTO rs9939609 risk allele (T > A) greatly varies between the populations, i.e., 0.19–0.45; however, in south-Asian ancestry, the frequency is higher, i.e., 0.47 (17).

There is a large body of evidence on the correlation between fat mass and advanced glycation end products (AGEs) in obese individuals (18–20). One of the hallmarks of obesity is the formation of a vicious cycle connecting fat mass, AGEs, and inflammation. AGEs bind to their receptors or RAGE, which in turn suppresses antioxidant rescue mechanisms, thereby increasing systemic inflammation (21).

AGEs are formed due to non-enzymatic reduction of sugars with proteins, some lipids, or nucleic acids (18, 22). AGEs formation has been associated with consumption of highly processed foods, cooking at high temperatures, or prolonged consumption of a high-protein diet (23–26). Accumulation of AGEs in the body has been associated with increased inflammation and oxidative stress, resulting in macro and microvascular complications (26–28). Among the different AGEs markers, Nε-carboxy-methyl-lysine (CML) has been frequently used as a biomarker of advanced glycation (29). Elevated levels of CML have been reported in subjects with obesity that may lead to inflammation and oxidative stress (23, 30). CML also interrelates with specific pro-inflammatory or anti-inflammatory receptors (20). As reported that higher intakes of both animal and vegetable protein have been associated with higher CML concentration (31). Moreover, in a short-term human intervention study, consumption of a high-protein (47%), low-calorie diet has shown an antiglycation effect (27). BMI and WC reduction are evident from a short-term protein diet intervention in the Japanese population, where BMI was reduced by 6.3% and waist circumference (WC) was reduced by 5.7% (27). High-protein diets have been recognized as effective for weight loss, but the effectiveness of these diets in individuals with genetic polymorphism still needs to be explored. The majority of the studies conducted on the association of diet-gene interaction have been conducted in European and Caucasian populations; therefore, examinations in other ethnic groups are warranted. High-protein diets have shown enhancement of satiety and reduction of fat mass in human intervention studies (8, 9). The effects of *FTO* polymorphisms on body weight can be modified by dietary macronutrients (32). The interaction between dietary protein intake and *FTO* genotype on weight loss has been studied in obese adults in the Spanish population, where carriers of the *FTO* rs9939609 risk allele (A) experienced significantly greater reductions in body mass and fat distribution in response to a high-protein diet compared with T allele homozygotes (33). The association of the rs9939609 polymorphism with obesity has been shown to be dependent on dietary macronutrient intake in several European cohorts. In a Spanish cohort of obese children, the consumption of high saturated fat intake has been linked to the rs9939609 SNP of the *FTO* gene. The risk allele carriers consuming more than 12.6% saturated fatty acids (of total energy) had an increased obesity risk compared with TT carriers (26). In the POUNDS LOST trial, participants with the FTO risk allele (A) on high-protein diets showed greater improvements in body composition and fat distribution compared to those on low-protein diets (34). Other studies have also shown the mitigating effect of dietary protein on the genetic risk associated with the *FTO* rs9939609 polymorphism. There is a strong association with CML and consumption of both animal and plant proteins. However, the main reason is not just the protein quantity but also the processing methods and food matrices. As one study showed that high protein intake led to an increase in levels of both CML and sRAGE, however, the CML/sRAGE ratio remained unaffected, which may be due to some compensatory mechanism. Another key aspect is the choice of cooking method, where prolonged dry heat applied during grilling/frying produces higher levels of CML compared with boiling, steaming, or extrusion (35). Positive effects of caloric restriction in mice have been attributed to enhanced lysine degradation and reduction in glycative stress. However, the same benefits are generally not replicated in the same way in humans, where reduced substrate due to CR is more likely to occur compared with protein modulation (36). Another reason for using a high-protein diet stems from the fact that evidence suggests preservation of muscle mass, which is a major CML reservoir. Therefore, although seemingly paradoxical or counterintuitive, a high-protein low-calorie diet is likely to reduce CML levels (37). Another important aspect of this study was to investigate the gene (FTO)–nutrient (high-protein low-caloric diet) in our population. There is already evidence showing that FTO plays a role in nutrient sensing (38), while reports suggest that the use of a high-protein diet can also protect against risk genotypes of FTO (39). Thus, the FTO genotype could modulate CML dynamics during protein-focused interventions. A decrease in IL6 has been shown in patients with metabolic syndrome undergoing dietary intervention (40).

Therefore, consumption of a high-protein diet may be an effective approach for weight loss in individuals with the rs9939609 carriers of the FTO gene by lowering food cravings and appetite (38). While many nutrigenomic studies have focused on obesity, there has been much less research on the interaction between diet and SNPs in obesity, particularly in Pakistani populations. Therefore, this study aims to assess the potential impact of a high-protein low-calorie (HPLC) diet on anthropometric indices across different variants of the FTO gene in overweight and obese individuals. In addition, it explores the potential impact of a high-protein with low-calorie (HPLC) diet on CML and IL-6 across different variants of the FTO gene in overweight and obese individuals. IL-6 was selected as a primary endpoint because it integrates inputs from adipose inflammation, CML-RAGE activation, and dietary modulation. Its responsiveness to protein quantity makes it ideal for evaluating the inflammatory outcomes of our intervention.

## 2 Materials and methods

### 2.1 Study design and sample size

This interventional study was conducted at the Institute of Basic Medical Sciences (IBMS), Khyber Medical University (KMU), Peshawar. The sample size for the intervention was calculated using OpenEpi software (version 3). Assuming a power of 80%, confidence interval of 95%, and by taking the mean ±SD of CML before (0.07 ± 0.017) and after intervention (0.06 ± 0.009). Almost 146 overweight and obese individuals were genotyped based on the Minor Allele Frequency of 0.1 for the FTO gene polymorphism (rs9939609). The Hardy–Weinberg Equilibrium (HWE) for three under-study genotypes (TT, AA, and AT) was calculated. The frequencies were not consistent with HWE, i.e., *p* < 0.05. The Hardy–Weinberg Equilibrium analysis for FTO rs9939609 showed inconsistent results, opposing previous findings. This discrepancy may be due to the small sample size and the same ethnicity of the study population, as Hardy–Weinberg equilibrium assumptions are more significant in larger, ethnically diverse populations (41).

### 2.2 Participant selection

Participants who were overweight and obese (both men and women), aged 18–50 years, and had a BMI of more than 25 kg/m^2^ were recruited. Participants with any history of diabetes, heart disease, impaired hepatic, renal, or thyroid function, and those suffering from psychiatric disorders/eating disorders were excluded from the study. In addition, participants using medication for weight loss and those who lost more than 5 lbs. in the last 3 months, pregnant and lactating women were also excluded from the study. Participants were recruited using posted flyers that were designed to include the study purpose and objectives. Furthermore, participants who met the inclusion criteria were briefed about the study, and a written consent form was signed.

### 2.3 Ethical approval and trial registration

The study was approved by the Institutional Review Board Ethics committee of the Institute of Basic Medical Sciences (IBMS) under registration number KMU/IBMS/IRBE/7th/2023/1209–20. The trial is registered at Clinicaltrials.gov under the registration number NCT06426017.

### 2.4 Group allocation and intervention

**Figure 1:**
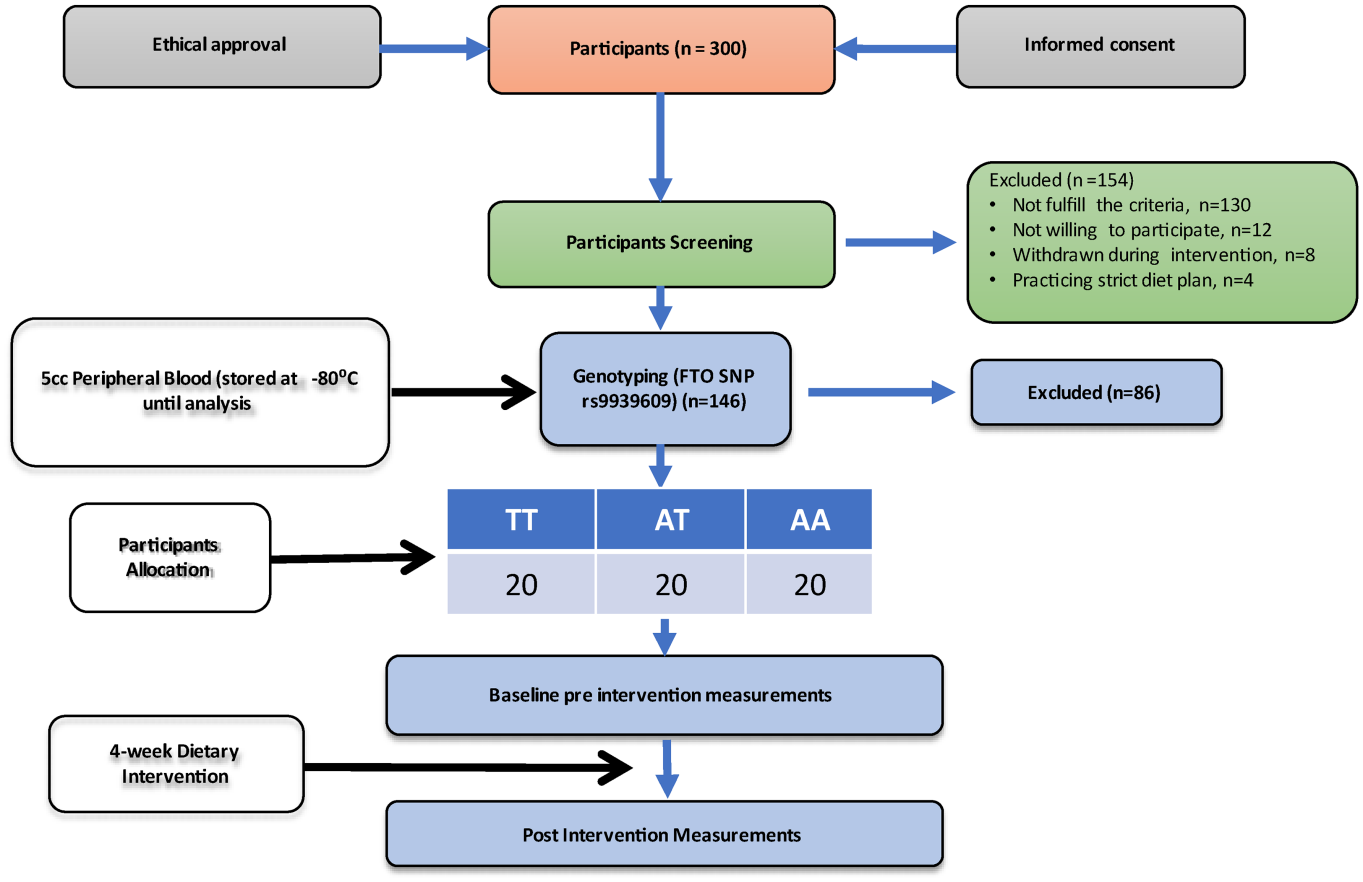
Study flow chart.

The overweight and obese participants recruited in this study were genotyped for the FTO gene polymorphism (rs9939609) using allele-specific PCR (Bio-Rad T100 Thermal Cycler). After genotyping, participants were categorized into three categories based on their genotypes into wild type allele (TT), homozygous for the minor allele (AA), and heterozygous allele (AT), respectively. A total of 60 participants were divided into three genotypes, i.e., (i) the wild type allele (TT; *n* = 20), (ii) homozygous for the minor allele (AA; *n* = 20), and (iii) heterozygous allele (AT; *n* = 20). All the groups received high high-protein (~100 g) and low-calorie (800 Kcal/day) diet for 4 weeks. Blood samples were taken at the start of the intervention (baseline) and after 4 weeks (endline). Figure 1 shows the experimental protocol.

### 2.5 Weight and anthropometric measurements

Height and weight were recorded using a digital electronic scale (HF-5664) and a Portstad Portable Stadiometer (HM200P), respectively. For weight measurements, participants were asked to remove outer heavy clothing and shoes to measure weight accurately. Participants were instructed to stand as straight as possible and position their head in Frankfurt line, with the ear canal aligned at the level of the cheekbone. They were then asked to take a deep breath and hold it while height was measured in centimeters. Weight and height measurements were used to calculate BMI as: BMI = weight (kg)/height (m^2^). The World Health Organization (WHO) criteria were used for classification of nutritional status as > 18.5 kg/m^2^ underweight, 18.5–24.9 kg/m^2^ normal, 25–29.9 kg/m^2^ overweight, and ≥ 30 kg/m^2^ obese. WC was measured by placing an inch tape around the middle of the bottom of the ribs and the top of the hip bone, just above the belly button. Hip circumference (HC) was measured by placing an inch tape around the widest part of the buttocks. Readings were taken in centimeters. Waist-to-hip ratio was calculated by dividing WC by HC.

### 2.6 Blood collection for DNA extraction and genotyping of the rs9939609 FTO gene polymorphism

**Figure 2:**
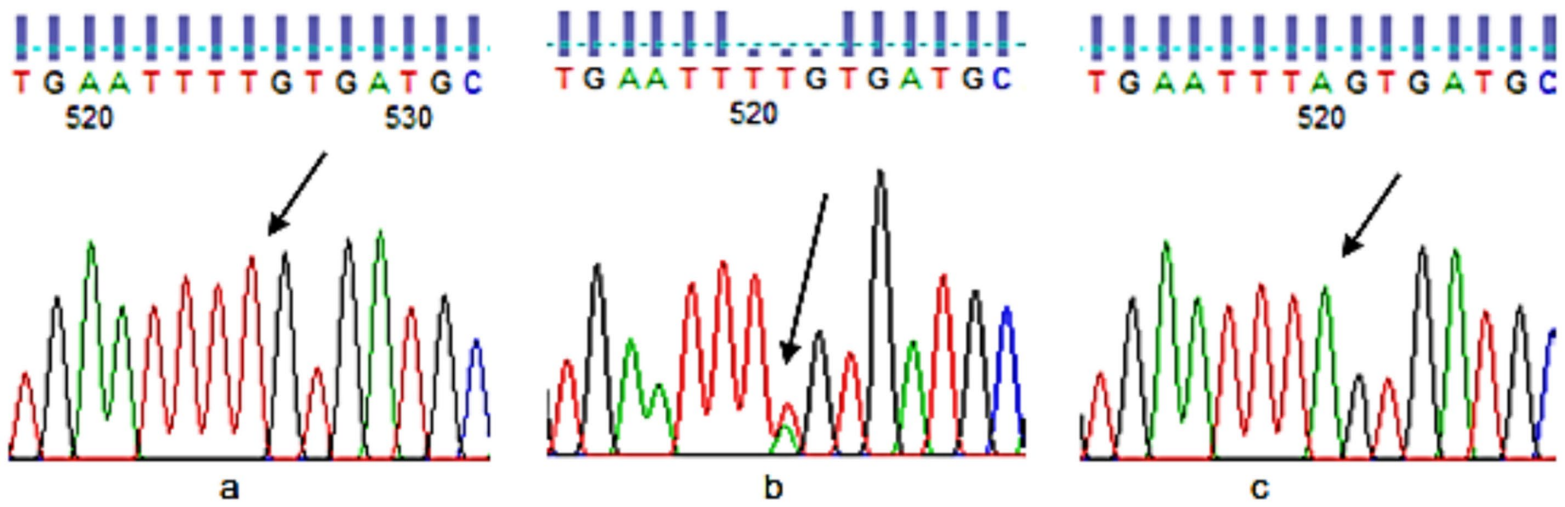
FTO rs9939609 genotypes sequencing **(a)**: TT, **(b)**: AT, and **(c)**: AA.

A whole blood (5 cc) sample was taken for DNA extraction from each participant with the help of a syringe. The upper arm was wrapped in a tourniquet. With an alcohol swab, the needle entry site was cleansed, and blood was gently drawn into the syringe. Blood was transferred into a prelabeled ethylene diamine tetraacetate (EDTA) tube. The plasma was removed after centrifugation at 4,000 rpm for 10 min. The resultant samples were stored at −80 °C for the extraction of DNA. The DNA was extracted from the samples using the manual technique of salting out introduced by Miller et al. (42). For direct sequencing of FTO-rs9939609, all the DNA samples were sent to Tsingke Biotech, China. The results were analyzed using Finch TV software. To determine the exact location of the SNP and the adjacent upstream and downstream nucleotide sequences, dbSNP was used. Color marks were used in the software to identify different nucleotides, i.e., Red for T, Green for A, Blue for C, and Black for G. However, some samples did not produce readable results, so they were genotyped in the lab using Allele-specific PCR (Bio-Rad T100 Thermal Cycler). Genotyping of FTO for SNP rs9939609 was done by directly amplifying the flanking region of the TAG SNPs and sequencing using the Sanger’s chain transmission method. Allele-specific PCR was performed for genotyping. SNP was selected from the FTO gene involved in obesity. Primers were designed using the NCBI website. The UCSC browser was used for attaining a longer sequence of the SNP rs9939609. Primers were designed through^1^ . Primer validity was checked against the Human Genome Browser of NCBI through BLAST (Figure 2).

### 2.7 Dietary intervention and meal preparation

A high-protein, low-calorie diet (HPLCD) was given to individuals for 4 weeks. A daily meal of 800 kcal was designed with 40–60% of energy from proteins, <30% energy from fats, and <20% energy from carbohydrates. Meals were carefully prepared with appropriate quantities using kitchen measuring tools (cups, spoons, and a weighing scale) for each ingredient, following healthy cooking methods and hygiene. Meals were carefully packed in disposable meal boxes labeled with participant ID and day. All the participants were requested to receive their meals from the clinical trial unit kitchen, Khyber Medical University. Meals were delivered to some participants living far away.

### 2.8 Biochemical assay

Blood was collected from all the participants at baseline and at the end of the intervention into prelabeled serum tubes. The samples were centrifuged at 4,000 rpm for 10 min and aliquoted into already labelled cryovials. Serum separated was stored at −80 °C until analysis for determination of serum CML and IL-6 levels. Serum CML and Serum IL-6 levels were determined using respective ELISA Kits (Human Carboxymethyl lysine (CML) BT Lab Kit, Cat No. E1413Hu; Human Interleukin 6, IL-6 BT LAB kit Cat. No. E0090Hu). Absorbance was measured at 450 nm using a microplate reader.

### 2.9 Statistical analysis

Data analysis was carried out using SPSS software for Windows. Data distribution was checked using the Shapiro–Wilk test. Continuous variables, such as anthropometric and biomarkers, were presented as mean ± standard deviation (SD). Frequency and percentage were used for categorical variables, such as sex and genotype. Hardy–Weinberg Equilibrium (HWE) was calculated using the Hardy–Weinberg calculator to determine the variation in genotype and allelic frequencies within the study population, where *p* > 0.05 was considered significant. Paired t-tests were applied to observe the differences in anthropometric measurements and biomarkers in AA, AT, and TT genotypes at baseline and endline of the HPLC diet intervention. Analysis of variance (ANOVA) with Bonferroni’s *post-hoc* analysis was performed to test the differences in baseline characteristics, anthropometric measurements, and biomarkers across the three genotypes of the rs9939609 polymorphism. Finally, analysis of covariance (ANCOVA) with Bonferroni’s *post-hoc* analysis was performed to test the differences of endline changes in anthropometric measurements and biomarkers across the three genotypes of the rs9939609 polymorphism after adjusting for covariates such as age and baseline values. A 𝑃-value of < 0.05 was considered statistically significant (Table 1).

**Table 1:** Calories and macronutrient distribution of HPLC diet for intervention.

Food	Amount	Calories (kcal)	Carbohydrates (g)	Fats (g)	Proteins (g)
Breakfast					
Boiled egg	2 Eggs	144	1	10	12
Milk/Tea without sugar	1 cup	150	11	8	9
Lunch					
Grilled chicken	2 Breast	302	-	6	60
Sauteed vegetables	Fillets 1 cup	120	8	1	8
Dinner					
Lentil curry	¼ cup	56	8	-	4
Grilled paneer	¼ cup	50	-	4	3
Whole wheat roti	½ roti (7″)	53	11	-	2
Total		875	39	29	98
		800	< 40 g	< 27 g	100 g

## 3 Results

### 3.1 Baseline characteristics

The mean age and height of the participants were 29.61 ± 6.92 years (range: 19–42) and 167.85 ± 9.19 cm (range: 135–185 cm), respectively, with no significant difference across the three genotypes of the FTO gene. The weight (*p* = 0.01), WC (*p* = 0.002), and WHR (*p* = 0.04) were significantly different among the three genotypes. The mean weight (95.74 ± 19.13 kg), WC (105.85 ± 14.55 cm), and WHR (0.93 ± 0.08) were higher for the homozygous AA risk allele. Moreover, there was no significant difference in the serum CML and IL-6 levels among the genotypes (Table 2).

**Table 2:** Baseline characteristics of the participants.

Characteristics		Mean ± SD/ n (%)			*p-*value
		TT (*n* = 20)	AA (*n* = 20)	AT (*n* = 20)	
Age (years)		31.33 ± 6.33	30.00 ± 7.54	27.40 ± 6.73	0.19
Sex	Male	8 (38.1)	14 (70)	12 (60)	0.11
	Female	13 (61.9)	6 (30.0)	8 (40.0)	
Height (cm)		166.19 ± 7.47	171.45 ± 8.40	166.00 ± 10.82	0.10
Weight (kg)		81.6 ± 11.38	95.74 ± 19.13	84.13 ± 11.03	**0.01**
BMI (kg/m^2^)		29.55 ± 3.673	32.46 ± 5.10	30.69 ± 4.78	0.13
WC (cm)		93.41 ± 7.21	105.85 ± 14.55	96.90 ± 10.80	**0.002**
HC (cm)		107.79 ± 9.02	113.36 ± 9.02	109.04 ± 7.38	0.10
WHR		0.87 ± 0.06	0.93 ± 0.08	0.89 ± 0.08	**0.04**
Glycation and Inflammatory Markers					
Baseline CML		2.47 ± 0.42	2.50 ± 0.44	2.53 ± 0.39	0.92
Baseline IL-6		1.86 ± 0.34	1.87 ± 0.38	1.86 ± 0.31	1.00

Data are n (%) or mean ± SD unless otherwise indicated. *p values were calculated using the X^2^ test for categorical variables and ANOVA for continuous variables. Boldface p values indicate statistical significance. BMI: Body mass index, WC: waist circumference, HC: hip circumference, WHR: waist to hip ratio, CML: carboxymethyl lysine, IL-6: interleukin-6.

### 3.2 Frequency of genotyping

Genotyping was done for 146 overweight and obese individuals for FTO rs9939609 to attain the desired number of genotypes (TT, AA, and AT). The percentages of homozygous TT, heterozygous AT, and homozygous AA were 42.5, 37.7, and 19.9%, respectively.

### 3.3 Effect of HPLC diet on anthropometric measurements of the participants among the three genotypes

Anthropometric characteristics of participants at baseline and after 4 weeks of intervention are shown in Table 3. After consumption of the HPLC diet, weight, BMI, WC, and HC significantly decreased (*p* < 0.05) in all three genotypes, except for WHR in the risk AA allele, by the end of the intervention.

**Table 3:** Effect of HPLC diet on anthropometric measurements of the participants among the three genotypes.

Parameter	Time	TT (*n* = 20)	AA (*n* = 20)	AT (*n* = 20)
Weight (kg)	Baseline	81.61 ± 11.38	95.74 ± 19.13	84.13 ± 11.03
	Endline	78.23 ± 11.03*	92.16 ± 17.83*	81.61 ± 10.77*
BMI (kg/m^2^)	Baseline	29.55 ± 3.67	32.46 ± 5.10	30.69 ± 4.78
	Endline	27.85 ± 4.02*	31.36 ± 4.83*	29.67 ± 4.54*
HC (cm)	Baseline	107.79 ± 9.02	113.36 ± 9.02	109.04 ± 7.38
	Endline	105.61 ± 9.32*	110.54 ± 7.92*	107.30 ± 7.31*
WC (cm)	Baseline	93.41 ± 7.21	105.85 ± 14.55	96.90 ± 10.80
	Endline	88.76 ± 6.90*	102.08 ± 13.37*	92.78 ± 10.90*
WHR	Baseline	0.87 ± 0.06	0.93 ± 0.08	0.89 ± 0.08
	Endline	0.85 ± 0.06*	0.92 ± 0.08	0.87 ± 0.09*

Values are expressed as Mean ± SD. BMI: Body mass Index, WC: Waist circumference, HP: Hip circumference, WHR: Waist-to-hip ratio. Paired sample *t*-test: ^*^P < 0.05 (Baseline vs 4 weeks).

A one-way ANOVA was conducted to compare percent changes in anthropometric indicators across three FTO genotypes (TT, AA, and AT) following the HPLC diet (Table 4). Percent BMI loss showed a statistically significant difference among the genotypes (*p* = 0.03). *Post-hoc* pairwise comparisons revealed that participants with the TT genotype experienced significantly greater reductions in BMI (5.86 ± 4.92%) compared to those with AA (3.23 ± 2.58%) and AT (3.25 ± 2.40%) genotypes, as indicated by different superscript letters (a ≠ b). For percent weight loss, although the TT group showed a higher mean reduction (4.14 ± 2.17%), the difference was not statistically significant (*p* = 0.27). No significant genotype differences were observed in WC loss, HC loss, or waist-to-hip ratio (WHR) loss (*p* > 0.05 for all). These findings suggest a potential genotype-dependent response to weight loss, particularly for BMI reduction, where TT genotype individuals responded more favorably. Participants with the TT genotype showed the greatest mean percent loss in body weight (4.13%), BMI (5.86%), and WC (−493.33%) compared with AA and AT genotypes. The AA genotype group showed the lowest reductions across most metrics, including WHR and WC. These descriptive findings suggest that genotype may influence responsiveness to weight loss interventions.

**Table 4:** Percent changes in weight loss, BMI Loss, and body composition parameters across FTO genotypes (TT, AA, AT).

Parameter	TT	AA	AT	*P-*value
% Weight Loss	4.14 ± 2.17	3.00 ± 2.74	3.50 ± 1.68	0.27
% BMI Loss	5.8563 ± 4.91771^a^	3.2306 ± 2.57940^b^	3.2548 ± 2.39582^b^	0.03*
% WC Loss	−493.33 ± 322.34	−344.48 ± 224.72	−429.79 ± 248.45	0.23
% HC Loss	−202.09 ± 267.98	−235.67 ± 216.54	−158.59 ± 149.56	0.54
% WHR Loss	−220.00 ± 327.07	−72.93 ± 368.16	−284.39 ± 283.86	0.13

Values are mean ± SD of the percent change of anthropometric measurements across genotypes. BMI: Body mass Index, WC: Waist circumference, HP: Hip circumference, WHR: Waist-to-hip ratio. ANOVA ^*^P < 0.05. Means with different letters along the row are statistically significant (ANOVA * *P* < 0.05).

### 3.4 Effect of HPLC diet on biomarkers of inflammation

Table 5 shows changes in serum CML and IL-6 levels. At the end of the intervention, no significant (*p* > 0.05) changes were observed in CML and IL-6 among all three genotypes.

**Table 5:** Effect of HPLC diet on biomarkers of inflammation among three genotype groups.

Parameter	Time	TT (*n* = 20)	AA (*n* = 20)	AT (*n* = 20)
CML (ng/ml)	Baseline	2.50 ± 0.44	2.47 ± 0.42	2.53 ± 0.39
	Endline	2.45 ± 0.43	2.50 ± 0.36	2.51 ± 0.32
IL-6 (ng/L)	Baseline	1.87 ± 0.38	1.86 ± 0.34	1.86 ± 0.31
	Endline	1.85 ± 0.35	1.85 ± 0.33	1.84 ± 0.33

Values are expressed as Mean ± SD. CML: Carboxymethyl lysine, IL-6: Interleukin 6. Paired sample *t*-test: ^*^P < 0.05 (Baseline vs. 4-weeks).

### 3.5 Comparative effect of HPLC diet on anthropometric parameters, CML, and IL-6 among the three groups

Table 6 shows the effect of the HPLC diet on anthropometric and biochemical parameters across the three FTO genotypes after adjustment for age as a covariate. After dietary intervention with the HPLC diet, weight (*p* = 0.02), BMI (*p* = 0.03), WC (*p* < 0.001), and WHR (*p* = 0.02) significantly reduced across all the genotypes. The risk allele AA had significantly higher weight, BMI, WC, and WHR compared with the TT allele. The improvement of all anthropometric parameters was statistically significant across the three genotype groups, with a significant difference between the homozygous risk allele and wild type TT. The effect size estimates also show a significantly larger effect of genotypes on weight (*η2* = 0.158), BMI (*η2* = 0.114), WC (*η2* = 0.235), and WHR (*η2* = 0.138). No significant differences were observed on CML (*p* = 0.26) and IL-6 (*p* = 0.37) levels across the three genotypes. The results are significant only for BMI (*p* = 0.05) when adjusted for baseline values.

**Table 6:** Comparative effect of HPLC diet on anthropometric parameters, CML, and IL-6 among the three groups.

Parameter at Endline	TT	AA	AT	*P*	*η2*
Weight (kg)	78.23 ± 11.03^b^	90.04 ± 15.52^a^	81.61 ± 10.77^ab^	**0.02**	0.158
BMI (kg/m^2^)	27.85 ± 4.02^b^	30.68 ± 3.86^a^	29.67 ± 4.54^ab^	**0.03**	0.114
WC (cm)	88.76 ± 6.90^b^	100.82 ± 12.45^a^	92.78 ± 10.90^ab^	**< 0.001**	0.235
HC (cm)	105.61 ± 9.32	109.83 ± 7.46	107.30 ± 7.31	0.19	0.057
WHR	0.85 ± 0.06^b^	0.92 ± 0.08^a^	0.87 ± 0.09^ab^	**0.02**	0.138
CML (ng/mL)	2.45 ± 0.43	2.50 ± 0.36	2.51 ± 0.32	0.26	0.045
IL-6 (ng/L)	1.85 ± 0.35	1.85 ± 0.33	1.84 ± 0.33	0.37	0.035

Values are mean ± SD. HC: Hip circumference, WC: Waist circumference, WHR: Waist-to-hip ratio, CML: Carboxymethyl Lysine, IL-6: Interleukin 6. One-way ANCOVA adjusted for age for differences across genotypes; Boldface P values indicate statistical significance (*P* < 0.05). Means with different letters along the row are statistically significant ( *P* < 0.05).

## 4 Discussion

The present study is the first one to evaluate the impact of HPLC diet intervention on weight loss and markers of advanced glycation and inflammation in the Pakistani overweight and obese population. The study demonstrated that the HPLC diet was effective in weight loss in both the risk allele and wild type of the FTO gene. The risk allele in both homozygous and heterozygous individuals of the FTO had a higher tendency for excess weight and adiposity. However, no effect was observed on the marker of advanced glycation and inflammation across the genotypes of the FTO genes before and after dietary intervention. To attain the desired number of genotypes (AA, TT, and AT), 146 overweight and obese individuals were genotyped for FTO rs9939609. The percentages of homozygous TT, heterozygous AT, and homozygous AA were 42.5, 37.7, and 19.9%, respectively. The FTO risk allele has been frequently reported in the European population (~ 42%) and least reported in the African population (~ 12%), accounting for 0.3 and 0.1% variation in BMI, respectively (43, 44). The rs9939609 polymorphism of the FTO gene has been widely associated with obesity in several European cohorts (32). In Asian populations, the FTO risk allele occurs with a frequency of 30%, accounting for 0.2% of the variation in BMI (45).

In the present study, no significant differences were observed in the mean age and height of the participants across the FTO genotypes. While the anthropometric measurements, such as weight, WC, and WHR, showed significant differences among the three genotypes of FTO, it was higher for the homozygous AA risk allele. In the present study, anthropometric measurements, such as weight, BMI, WC, HC, and WHR, were higher for the homozygous (AA) and heterozygous (AT) risk alleles compared with wild type TT alleles of the FTO genes. These results are in line with a study conducted in the Italian population on the association of FTO SNP rs9939609, showing higher anthropometric measurements and body composition in carriers of the risk allele A (46). Studies have shown that the FTO risk allele is associated with a 0.25–0.41 kg/m^2^ increase in BMI and 20–40% increased risk of obesity (43). Individuals with a homozygous risk allele of FTO are likely to be 3 kg heavier than those without the risk allele (13).

In the present study, with dietary intervention with the HPLC diet, weight, BMI, WC, and WHR, except for HC and for the risk allele AA, significantly decreased in all three genotypes. This interaction between dietary protein intake and *FTO* genotype on weight loss has been studied in obese adults in a Spanish population (29). Their results showed that those carriers of the FTO rs9939609 risk allele (A) experienced significantly greater reductions in body mass and fat distribution in response to a high-protein diet compared to T allele homozygotes, while this effect of *FTO* genotype was not observed in the low-protein treatment group (29). These findings suggest that the *FTO* variants interact with dietary protein intake to influence measures of body weight in individuals of diverse ethnocultural backgrounds, including our population as well. The effects of several *FTO* polymorphisms on body weight can be modified by various dietary parameters (32). The association between the FTO rs9939609 polymorphism and obesity has been shown to depend on dietary macronutrient intake in several European cohorts. In a Spanish cohort of children and adolescents, an interaction was observed between the consumption of SFA (percentage of total energy) and PUFA: SFA ratio and obesity risk linked to the rs9939609 SNP of the FTO gene. They reported that the risk allele carriers consuming more than 12.6% SFA (of total energy) had an increased obesity risk compared with TT carriers. In a similar way, A allele carriers with an intake ratio lower than 0·43 PUFA: SFA presented a higher obesity risk than TT subjects (32).

Recently, there has been increased interest in determining whether dietary macronutrient composition interacts with variation in *FTO* to influence measures of body weight. In the POUNDS LOST trial, a 2-year randomized weight loss intervention, subjects with the risk allele (A) placed on high-protein diets experienced greater positive changes in body composition and fat distribution compared with the low-protein diet group. No such effect of protein was observed among carriers of the T allele (30). These results suggest that dietary protein can mitigate the genetic risk associated with *FTO*. A recent study of weight loss has also reported greater weight loss and improvement in metabolic parameters in individuals with the *FTO* risk allele when placed on a high-protein diet (29). They identified an association between the rs9939609 polymorphism and higher dietary protein intakes, which our study has also reported.

After dietary intervention with the HPLC diet, weight, BMI, WC, and WHR significantly reduced across all the genotypes after adjustment for age. The risk allele AA had significantly higher weight, BMI, WC, and WHR compared with the TT allele. The improvement of all anthropometric parameters was statistically significant across the three genotype groups, with a significant difference between the homozygous risk allele AA and the wild-type TT. The effect size estimates also explained significant genetic variation of the FTO gene in weight, BMI, WC, and WHR. When the results were adjusted for baseline values, there was only a significant change in BMI. A study on the FTO gene’s impact on BMI and WC found that East Asians homozygous for the rs1558902 risk allele (A) had higher BMI and WC compared with T allele carriers, a pattern not observed in Caucasians or South Asians. Among East Asians, a significant interaction between FTO and protein intake was seen in those with low protein intake (≤ 18% of total energy), and the rs1558902 risk allele had significantly higher BMI and WC. These associations disappeared with higher protein intake (> 18%). Additionally, East Asians had a higher animal-to-plant protein ratio than Caucasians and South Asians (*p* < 0.05). This suggests high protein intake may protect against the effects of FTO risk variants on BMI and WC (39). BMI and WC reductions are evident from a short-term protein diet intervention in the Japanese population, where BMI was reduced by 6.3% and WC was reduced by 5.7% (27).

In the present study, no significant difference was observed in the serum CML and IL-6 levels among the three FTO genotypes at baseline and at the end of intervention. These findings are consistent with a Danish study in men that reported no association between *FTO* rs9939609 and IL-6 levels (47). This suggests that the FTO variant may not directly influence systemic inflammation as measured by IL-6. The lack of a statistically significant reduction in CML and IL-6 levels following HPLC dietary intervention may reflect the complexity of dietary modulation of inflammatory and glycation markers. For instance, a 2-month low-calorie (1,200 kcal/day, 47% protein) dietary intervention in the Japanese population demonstrated a reduction in AGEs, including CML (27). This discrepancy may be due to differences in calorie restriction levels, length of intervention, baseline metabolic status, or population-specific dietary patterns. Evidence from controlled feeding trials also highlights that protein quantity and source may play a role in modulating inflammatory responses. A three-week hypocaloric study in individuals with obesity found that a low-protein diet (10% of total energy) led to a more pronounced reduction in IL-6 compared with a high-protein diet (30% of total energy), despite similar energy restriction (48). Their study demonstrated that the type of protein may also play a role, as they used both animal and plant-based proteins. Furthermore, a study in Caucasian adults with obesity and metabolic syndrome showed that energy-restricted diets with plant-based protein reduced inflammatory markers, including IL-6, to a greater extent than diets with animal-based protein (49). These findings suggest that both the amount and the type of dietary protein could influence inflammatory responses, potentially explaining the variability in results across different studies. Taken together, our findings add to the growing body of literature indicating that the effects of macronutrient composition on inflammatory and glycation biomarkers are influenced by multiple factors, including dietary protein source, degree of energy restriction, intervention duration, and baseline metabolic status. Future research with larger sample sizes, longer intervention periods, and direct comparisons of protein sources may help clarify these relationships.

The present study has some limitations. This study analyzed only one SNP of the *FTO* gene, i.e., rs9939609 was the common polymorphism reported for our population in the literature, and so other genetic variants could also be linked to obesity and other metabolic parameters. Several uncontrolled factors, such as epigenetic influences, hormonal status, and physical activity levels, could affect our results. In addition, the absence of a control group without dietary intervention may introduce bias. Some findings need to be reinvestigated through studies involving high-protein low-calorie diets with longer intervention periods and large populations with different ethnicities.

## 5 Conclusion

This study concludes that a high-protein, low-calorie diet modifies the association between the FTO rs9939609 genotype and anthropometric measurements in our population. These findings suggest that high dietary protein intake may protect against the obesogenic effects of the rs9939609 genotype of the *FTO* gene. These findings support a link between *FTO* genotype, protein intake, and anthropometric measurements. Investigating the underlying mechanisms driving gene-diet interaction represents a crucial direction for future research. Furthermore, subsequent studies should aim to assess the efficacy and applicability of this nutritional strategy within personalized weight loss interventions.

## Data Availability

The original contributions presented in the study are publicly available. This data can be found here: https://www.ncbi.nlm.nih.gov/nuccore/PX434724.

## Footnotes

^1^ https://www.ncbi.nlm.nih.gov/tools/primer-blast/
